# Novel anti-virulence strategy against *Helicobacter pylori* using *Lactiplantibacillus plantarum* cell-free supernatants

**DOI:** 10.3389/fmicb.2026.1881796

**Published:** 2026-07-03

**Authors:** Nazly Reda El-Sayed, Reham Samir, Lina J. M. Abdel-Hafez, Kareem Ibrahim, Reem Binsuwaidan, Heba Selim, Mohammed A. Ramadan

**Affiliations:** 1 Department of Microbiology and Immunology, Faculty of Pharmacy, October 6 University, Giza, Egypt; 2 Department of Microbiology and Immunology, Faculty of Pharmacy, Cairo University, Cairo, Egypt; 3 Department of Microbiology and Immunology, Faculty of Pharmacy, Egyptian Russian University, Cairo, Egypt; 4 Department of Pharmaceutical Sciences, College of Pharmacy, Princess Nourah bint Abdulrahman University, Riyadh, Saudi Arabia; 5 College of Pharmacy, AlMaarefa University, Diriyah, Riyadh, Saudi Arabia; 6 Research Center, Deanship of Scientific Research and Post-Graduate Studies, AlMaarefa University, Diriyah, Riyadh, Saudi Arabia

**Keywords:** *cagA*, *Helicobacter pylori*, *Lactiplantibacillus plantarum*, LC MS/MS, urease, *vacA*

## Abstract

*Helicobacter pylori* is one of the most prevalent pathogenic microorganisms worldwide. The treatment of *H. pylori* infections is challenging due to the increasing antibiotic resistance. Therefore, alternative therapies are crucial for addressing these issues. This study aimed to investigate alternative approaches to combat *H. pylori* infection using lactic acid bacteria. Nineteen LAB isolates exhibited strong tolerance to simulated gastric acid and bile salts, as well as notable auto-aggregation and co-aggregation capabilities. Among them, isolates L20, L22, and L40 showed superior performance. Their neutralized cell-free supernatants (n-CFSs) significantly inhibited *H. pylori* urease production by 69, 66, and 67%, respectively, and reduced biofilm formation by 88, 82, and 80%, respectively. Molecular identification revealed that isolates L20 and L40 belonged to *Lactiplantibacillus plantarum*, while L22 was identified as *Limosilactobacillus fermentum*. Furthermore, the n-CFS of *L. plantarum* L20 markedly downregulated the expression of *H. pylori* virulence genes, including *vacA*, *cagA*, *ureA*, and *babA,* with fold reductions of 79, 68, 90, and 96%, respectively. *In vivo*, treatment with *L. plantarum* L20 n-CFS resulted in 1.28 log reduction in *H. pylori* colonization (*p* < 0.001). Proteomic analysis further identified metabolically active proteins associated with catalytic and secondary metabolite pathways, supporting their mechanistic role in anti-*H. pylori* activity. These findings highlight the potential of LAB-derived n-CFS, particularly *L. plantarum* L20, as a promising antivirulent strategy against *H. pylori* infections.

## 1 Introduction

*Helicobacter pylori* has been classified as a group 1 carcinogen, providing compelling evidence of its involvement in the development of gastric cancer and certain lymphomas (IARC, 1994). *H. pylori* is implicated in the infection of more than 50% of the global population. Even though many individuals remain asymptomatic, 10–20% of them develop peptic ulcers, and 1–2% progress to gastric cancer or mucosa-related diseases (Brawner et al., 2014; Espinoza et al., 2018; Pucułek et al., 2018; Venerito et al., 2018). The incidence of *H. pylori* infection in developing countries has reached its upper limit (up to 90%), compared to that in developed countries (Al-Fakhrany and Elekhnawy, 2023). This may be attributable to disparities in socioeconomic status and lifestyle (Hooi et al., 2017). According to the available epidemiological data, *H. pylori* prevalence is highest in Africa, followed by Asia and Europe, and lowest in the Americas and Oceania (Liu et al., 2023). Egypt bears a particularly high burden of *H. pylori* infection, with reported prevalence rates ranging from 13 to 72% in children and from 26 to 90% in adults (Alboraie et al., 2019). Several studies have revealed that Egypt, alongside Libya and Nigeria, exhibits some of the highest infection rates in Africa, reaching up to 90% in certain populations (Liu et al., 2023). Moreover, the substantial heterogeneity in infection prevalence across the Middle East and North Africa region, ranging from 36.8 to 94% in adults, underscores the urgent need for localized research to develop effective therapeutic strategies (Alsulaimany et al., 2020).

*H. pylori* is a Gram-negative, spiral-shaped, microaerophilic bacterium whose flagella confer high motility, enabling it to penetrate the gastric mucosa. The numerous virulence factors of this bacterium produce a variety of evasive mechanisms that facilitate invasion, survival, colonization, and induction of additional inflammation in the gastric mucosa (Baj et al., 2021; Vital et al., 2022).

This bacterium can survive and colonize the human gut in harsh environments. A key factor behind these is the virulence factor urease (Sharndama and Mba, 2022). In addition, *H. pylori* produces adhesion proteins, including sialic acid-binding adhesin (SabA) and blood group antigen-binding adhesin (BabA). The *babA* and *sabA* genes are essential for *H. pylori’s* pathogenicity and colonization (Quintana-Hayashi et al., 2018). *H. pylori* initiates inflammatory responses through the expression of two major toxins, vacuolating cytotoxin A (VacA) and cytotoxin-associated antigen A (CagA), which interact with host epithelial and immune cells (Baj et al., 2021). The production of reactive oxygen species in epithelial cells by *H. pylori* has the potential to degrade DNA, indicating its implication in gastritis and gastric cancer (Stent et al., 2018). Additionally, biofilm formation may be essential for *H. pylori* long-term colonization (Hathroubi et al., 2018; Hathroubi et al., 2020). Biofilms serve as a barrier for bacterial cells and possess the capability to resist many antibiotics and the immune system, rendering their eradication a demanding undertaking (Zhang et al., 2023).

The conventional treatment for *H. pylori* infections typically involves a proton pump inhibitor combined with two antibiotics (clarithromycin, levofloxacin, or amoxicillin). Nonetheless, the escalating prevalence of drug resistance in *H. pylori* has restricted the consumption of antibiotics, rendering the elimination of *H. pylori* a significant obstacle (Hathroubi et al., 2018). To combat this issue, quadruple therapy was developed as an alternative treatment strategy (O'Connor et al., 2020). However, accumulating evidence indicates that resistance to commonly prescribed antibiotics now exceeds 30% globally (Ho et al., 2022; Boyanova et al., 2023; Salahi-Niri et al., 2024; Zhou et al., 2024). A 2024 meta-analysis reported global pediatric resistance rates of 32.6% for clarithromycin, 35.3% for metronidazole, and 13.2% for levofloxacin across all World Health Organization (WHO) regions (Salahi-Niri et al., 2024). In adults, resistance to clarithromycin and levofloxacin reaches up to 40% in various regions (Zhou et al., 2024). Notably, the resistance rates in Egypt appear to be markedly higher. A 2022 single-center study involving Egyptian adults reported a 100% resistance rate to metronidazole, with clarithromycin and levofloxacin resistance rates of 40 and 20%, respectively (Metwally et al., 2022). These investigations emphasize the growing global challenge of *H. pylori* antimicrobial resistance and underscore an urgent need for novel therapeutic strategies to combat this resistance and enhance the eradication of *H. pylori*, particularly in regions with high resistance prevalence, such as Egypt (Suzuki et al., 2019).

Recently, probiotics have garnered significant attention as a strategy to combat multidrug-resistant pathogens (Ayala et al., 2014). Numerous studies have demonstrated that probiotics positively influence human health by modulating gastrointestinal microbiota, enhancing immune responses, and inhibiting pathogenic microorganisms (Un-Nisa et al., 2022; Zaib et al., 2024).

Lactic acid bacteria (LAB), including *Lactobacillus* spp., have been widely investigated for their potential activity against *H. pylori* infection (Hong et al., 2023). LAB-derived cell-free supernatant (CFS) is the extracellular fraction obtained after the removal of bacterial cells and contains bioactive metabolites, including organic acids, peptides, and bacteriocins (Crowley et al., 2013; Binda et al., 2020). Unlike live probiotics, CFS can exert antimicrobial and anti-virulence effects without safety concerns related to viable bacterial cell administration and may diffuse more efficiently through the gastric mucus layer, making it particularly suitable for immunocompromised hosts (Xia et al., 2025). Given the rising antibiotic resistance and treatment limitations, CFS represents a promising non-antibiotic strategy for attenuating *H. pylori* virulence. Previous studies have shown that CFS can affect *H. pylori* through the organic acids, which reduce gastric pH and inhibit urease activity, as well as other antimicrobial compounds that interfere with bacterial growth and mucosal adherence (Lim, 2014; Saxena et al., 2020; Do et al., 2022). Despite investigations into the mechanisms of action of LAB-derived CFS, the precise underlying mechanisms remain largely unknown.

The primary objective of this study was to evaluate the anti-virulence potential of the neutralized cell-free supernatant (n-CFS) derived from *Lactobacillus* spp. against *H. pylori*. By neutralizing the supernatant to pH 6.5, we specifically targeted the non-acidic bioactive metabolites and proteins to discover anti-virulence therapeutic agents. We aimed to characterize these effects through *in vitro* biofilm, urease inhibition, and virulence gene downregulation, and to assess colonization reduction using an *in vivo* animal model. We also investigated the different LAB mechanisms against *H. pylori* using a comprehensive proteome analysis.

## 2 Materials and methods

### 2.1 Bacterial strains and culture conditions

A standard strain of *H. pylori* (ATCC 43504) was generously supplied by the Clinical Pathology Department, Faculty of Medicine, Cairo University, Egypt. *H. pylori* was cultivated on Columbia agar containing 7% defibrinated horse blood and selective DENT supplement (Oxoid, United Kingdom), and placed in an anaerobic jar (Merck, Germany) under anaerobic conditions at 37 °C for 72 h. The strain was stored in Brucella broth (BB) containing 20% glycerol at −80 °C.

### 2.2 Sample collection and isolation of lactic acid bacterial isolates

Samples were obtained from diverse dairy products, including fresh natural cow and buffalo milk, homemade yogurt, and cheese. A sufficient amount of sample was inoculated into De Man-Rogosa-Sharpe (MRS) broth and incubated for 48 h at 37 °C. Afterwards, 10-fold serial dilutions were prepared and added to MRS agar medium containing 0.01% (w/v) L-cysteine hydrochloride and 0.5% calcium carbonate. All plates were incubated at 37 °C for 48 h under microaerophilic conditions, and only colonies surrounded by a clear zone as a result of the solubility of CaCO_3_ in the acid produced were selected for further examination (Somashekaraiah et al., 2019; Leska et al., 2022).

### 2.3 Characterization of lactic acid bacterial isolates

The identification of LAB isolates was done according to their biochemical and phenotypic characteristics, including Gram staining, catalase and oxidase tests, bile salt hydrolase activity, and culture characteristics. Physiological tests encompassed the ability of the isolates to proliferate in the presence of sodium chloride (3 and 5% (w/v)). All isolates were catalase-negative, oxidase-negative, and Gram-positive bacilli with a morphology resembling that of these isolates were categorized as potential LAB isolates (Todorov et al., 2016).

### 2.4 Hemolytic activity analysis

Overnight LAB cultures were plated on blood agar media containing 7–10% horse blood and incubated for 48 h at 37 °C. The presence of zones surrounding the colonies was assessed. Clear, greenish, and no zones were recorded for *β*-hemolytic, *α*-hemolytic, and *γ*-hemolytic activities, respectively. Only isolates with gamma hemolysis were selected, whereas those with beta or alpha hemolysis were discarded because they were classified as pathogenic organisms (Bazireh et al., 2020).

### 2.5 Physiological characteristics of lactic acid bacterial isolates

A total of 119 LAB isolates were initially screened for probiotic parameters, including acid survival at pH 2.0, bile salt tolerance, auto-aggregation, and co-aggregation with *H. pylori*. Strains exhibiting superior overall performance across these criteria were selected for further analysis. The antimicrobial susceptibility profiles of the LAB isolates demonstrating superior probiotic activity were then determined. Subsequently, these strains were evaluated for their antimicrobial and anti-virulent activities against *H. pylori* (Somashekaraiah et al., 2019; Binda et al., 2020; Merenstein et al., 2023).

#### 2.5.1 Tolerance to bile salts

The tolerance of the LAB isolates to bile salts was investigated using the method outlined by Bazireh et al. (2020). Overnight cultures of the LAB isolates were inoculated into MRS broth containing 0.3% (w/v) oxgall (HiMedia, India). The culture media were incubated under microaerophilic conditions for 4 h at 37 °C. MRS broth devoid of oxgall served as a negative control. Finally, the optical density (OD) of the cultures was measured at a wavelength of 600 nm, and the percentage of growth inhibition was calculated using the following equation: Growth inhibition (%) = $\frac{\mathrm{Ay}-\mathrm{Ax}}{\mathrm{Ax}}\times 100.$ Where Ay is the absorbance of the MRS broth containing oxgall, and Ax is the absorbance of the control.

#### 2.5.2 Acid tolerance in simulated gastric juice

The survival of LAB isolates in simulated gastric juice was evaluated as described by Zhou et al. (2022). LAB isolates were inoculated into MRS broth containing L-cysteine and incubated for 24 h at 37 °C. The bacterial suspension was centrifuged at 8000 rpm for 10 min, and the pellets were resuspended in phosphate-buffered saline (PBS). An aliquot (100 μL) of the cell suspension was cultured in an artificial gastric solution containing 1 g/L of pepsin. The pH of this solution was adjusted to 2.0 using 1 N HCl, while the solution (pH 6.5) without the addition of gastric solution served as a negative control. The samples were subsequently kept in an incubator shaker for 3 h at 37 °C to mimic the peristaltic motion. Samples were then streaked on MRS agar and incubated under microaerophilic conditions after 0 and 3 h of incubation. The number of viable cells in each isolate was counted using the plate count method. The experiments were conducted in triplicate, and the number of viable cells was expressed as log CFU/mL for each tested isolate. The survival percentage was calculated as the percentage of surviving cells relative to the initial viable cells, using the subsequent formula: Survival (%) = (Log CFU of surviving cells)/(Log CFU of initial viable cells) × 100.

#### 2.5.3 Auto-aggregation of lactic acid bacteria

The auto-aggregation capability of the LAB isolates was assayed as reported by Somashekaraiah et al. (2019). The isolates were centrifuged at 10,000 rpm after an overnight culture. The pellets were subsequently collected, washed twice, and resuspended in PBS. The initial OD was adjusted to 0.50 ± 0.05 at 600 nm and incubated anaerobically at 37 °C. The absorbance of the upper layer was quantified at 600 nm at time intervals of 0, 1, 2, 4, and 24 h. Higher percentages reflect a greater auto-aggregation ability. The auto-aggregation percentage was determined using the subsequent equation: Auto-aggregation (%) = (A0 – At) / A0 × 100. Where A0 represents the OD of LAB isolates at 0 h, and At represents the OD of LAB isolates at 1, 2, 4, and 24 h.

#### 2.5.4 Co-aggregation of lactic acid bacteria with *Helicobacter pylori*

The co-aggregation ability was assessed using the method outlined by Sun et al. (2018). Overnight cultures of LAB isolates and *H. pylori* were centrifuged at 10,000 rpm at 4 °C for 10 min. The cells were harvested, washed twice with PBS, and resuspended in PBS. The initial OD of each isolate was adjusted to 0.50 ± 0.05 at 600 nm. Equal volumes of LAB isolates and *H. pylori* cell suspensions were mixed, vortexed for 10 s, and incubated at 37 °C. The tubes with each isolate alone were considered controls. At different time intervals, the absorbance of the upper suspension layer was measured at 600 nm. The co-aggregation rate was quantified using the following formula: Co-aggregation (%) = ((Ax + Ay) – 2Axy) / (Ax + Ay) × 100. Wherein Ax represents the OD of the LAB isolates at 0 h, Ay represents the OD of *H. pylori* at 0 h, and Axy represents the mixture OD at 1, 2, 4, and 24 h.

### 2.6 Antibiotic susceptibility

The antibiotic susceptibility of the selected LAB isolates was determined using the Kirby-Bauer disc diffusion method (Somashekaraiah et al., 2019). The 0.5 McFarland standard was prepared from an overnight culture of LAB isolates and spread onto MRS agar plates. Antibiotic disks were aseptically placed on the surface of each inoculated plate under anaerobic conditions at 37 °C for 48 h. After incubation, the diameters of antibiotic inhibition zones (mm) were measured and recorded. The results obtained from three independent experiments were used to classify isolates as resistant (R), intermediate (I), or sensitive (S) to a particular antibiotic, based on CLSI standards from three independent experiments (CLSI, 2020).

Antibiotic disks were manufactured by HiMedia (India) and included ampicillin (10 μg/disc), cefotaxime (30 μg/disc), clindamycin (2 μg/disc), erythromycin (15 μg/disc), gentamicin (10 μg/disc), kanamycin (30 μg/disc), penicillin (10 μg/disc), rifampicin (5 μg/disc), streptomycin (10 μg/disc), tetracycline (30 μg/disc), trimethoprim (25 μg/disc), and vancomycin (30 μg/disc).

### 2.7 Screening for antimicrobial activity of lactic acid bacteria supernatants

#### 2.7.1 Preparation of the cell-free supernatant

CFSs of LAB isolates were prepared using the following protocol, outlined by Apiwatsiri et al. (2021). Thus, the isolates were inoculated into MRS broth at an equivalent inoculum size of 1.5 × 10^8^ CFU/mL and placed under anaerobic conditions at 37 °C for 48 h. Subsequently, the cells were separated by centrifugation at 10000 rpm at 4 °C for 10 min. The resulting CFSs were divided into two fractions; the first one was kept at its initial acidic pH. While the remaining CFS (n-CFS) was adjusted to pH 6.5 using 1 N NaOH to eliminate the presumed effect of organic acids. The CFSs were then sterilized through bacterial syringe filters with a pore size of 0.22 μm. Finally, both supernatants were stored at −80 °C for further investigation.

#### 2.7.2 determination of antibacterial activity of lactic acid bacteria supernatants

The antibacterial activity of the LAB supernatants was examined using the agar diffusion method against standard cultures of *H. pylori*. The *H. pylori* culture was streaked on Columbia agar containing 10% sheep blood, and the wells were filled with 50 μL CFSs or n-CFSs. The plates were then incubated at 37 °C for 72 h under microaerophilic conditions. The inhibition zone around the wells was used to measure the antimicrobial activity, which was expressed as the average of the inhibition diameter (mm), with diameters ≥ 10 mm indicating antibacterial activity (Sircar and Mandal, 2023).

### 2.8 Effect of neutralized lactic acid bacteria supernatants on urease activity of *Helicobacter pylori*

The effect of n-CFS of LAB isolates on the urease activity of *H. pylori* was assayed using the phenol red method (Sun et al., 2018; Zhou et al., 2022) with some modifications. Fresh *H. pylori* culture was resuspended in BB medium supplemented with 10% heat- inactivated fetal bovine serum (FBS) and adjusted to 1.5 × 10^8^ CFU/mL. In a 96-well microtiter plate, the wells were filled with 40 μL of the *H. pylori* suspension with 10 μL of n-CFS of each isolate. The positive controls consisted of 10 μL of MRS broth (pH 6.5) and 40 μL of *H. pylori* suspension. Under microaerophilic conditions, plates were placed at 37 °C for 48 h. Afterwards, each well was loaded with 150 μL aliquot of urea solution, and the absorbance was determined at 590 nm. The test was done in triplicate, and the urease activity inhibition was evaluated using the following equation:


$$\text{Urease inhibition}\;(\%)=[1-\frac{(A_{c}-A_{t)}}{A_{c}}]\times 100.$$

### 2.9 Effect of neutralized lactic acid bacteria supernatants on biofilm formation of *Helicobacter pylori*

The influence of 50 and 25% of n-CFS on the formation of *H. pylori* biofilm was evaluated using the crystal violet (CV) microtiter plate method with some modifications (Apiwatsiri et al., 2021; Krzyżek et al., 2021; Abou Elez et al., 2023). After cultivating *H. pylori* on Columbia blood agar plates for 72 h at 37 °C under microaerobic conditions, the bacterial suspension was adjusted to 1.5 × 10^8^ CFU/mL in BB medium supplemented with 2% (v/v) FBS and 2% glucose. Subsequently, a volume of 100 μL of *H. pylori* suspension was added to a 96-well microtiter plate, followed by 100 μL of n-CFS at different concentrations. Microtiter plates were placed in an incubator at 37 °C for 48 h under microaerophilic conditions. Afterwards, the planktonic cells were removed, and the attached cells were carefully washed three times with sterile PBS. The wells were then allowed to dry at room temperature and stained with 200 μL of 0.1% CV (Sigma Aldrich) for 15 min. The dye adsorbed in the *H. pylori* biofilms was extracted by adding 200 μL of 95% ethanol, and biofilm formation was estimated by measuring the absorbance at 590 nm using a microplate reader. The biofilm inhibition assay was conducted in triplicate, and biofilm inhibition (%) was calculated as((Ax − Ay)/Ax) × 100. Where (Ay) represents the absorbance of wells containing *H. pylori* in contact with varying concentrations of LAB supernatants, and (Ax) represents wells containing *H. pylori* without LAB supernatants.

### 2.10 Molecular identification of selected lactic acid bacterial isolates

The most potent LAB isolates were selected as candidates for molecular identification using *16S rRNA* gene sequencing. The QIAquick PCR extraction kit (Qiagen, United States) was used to extract the genomic DNA from the LAB isolates. PCR was conducted using *16S rRNA* universal bacterial primers 16S-27F (5’AGAGTTTGATCCTGGCTCAG 3’) and 16S-1492R (5’TACGGTTACCTTGTTACGACTT 3’). The PCR reaction was carried out in a total volume of 25 μL, consisting of 12.5 mL EmeraldAmp GT PCR Master Mix (2X)(Takara, Japan), 1 μL forward and reverse primers (20 μM), 6 μL DNA template, and 4.5 mL PCR deionized water. PCR amplification was performed for 40 cycles in a thermocycler (Applied Biosystems, USA) as follows: initial denaturation for 5 min at 95 °C, followed by 35 cycles of denaturation for 1 min at 95 °C, annealing for 1 min at 55 °C, and elongation for 2 min at 72 °C. The final elongation step was performed for 10 min at 72 °C. The amplified products were checked using 1% agarose gel electrophoresis. The PCR products were sequenced, and the *16S rRNA* gene sequences were checked for similarities using the BLAST algorithm. Finally, phylogenetic analysis was performed using the MEGAX software.

### 2.11 Determination of the growth curve of *Helicobacter pylori* treated with LAB supernatants

The growth curve was constructed to determine the appropriate diluent of n-CFS of LAB isolates that permitted the growth of *H. pylori* without any adverse effects on its growth rate over a period of 24 h (Hasna et al., 2023). A pre-culture of *H. pylori* was diluted to 0.5 McFarland in BB medium containing 10% FBS and incubated in the absence (negative control) or presence of varying dilutions of n-CFS (non-diluted, 50, 25, and 12.5%) in 96-well plates. The culture plate was then placed under microaerophilic conditions at 37 °C for 24 h, and the OD was measured using a microtiter plate reader (Tecan, Austria) at time intervals of 0, 2, 4, 8, 12, and 24 h of exposure at a wavelength of 600 nm. The OD curve was constructed from the average values obtained from three distinct experiments.

### 2.12 Effect of lactic acid bacterial supernatant on virulence gene transcription of *Helicobacter pylori*

*H. pylori* was subcultured in BB medium supplemented with 10% FBS and incubated at 37 °C for 24 h under a microaerophilic environment. Fresh cultures equivalent to 1.5 × 10^8^ CFU/mL were prepared in three tubes and treated with n-CFS of *Lactiplantibacillus plantarum* 20 (tube 1), n-CFS of *Limosilactobacillus fermentum* 22 (tube 2), and without CFS (tube 3) as a negative control. The tubes were centrifuged for 10 min at 10000 rpm at 4 °C, and the resulting cells were washed three times with sterile PBS. Total RNA was extracted using the RNeasy Mini Kit (Qiagen, United States). RNA quality and purity were assessed prior to cDNA synthesis using a Nano-Drop spectrophotometer (ThermoFisher, Germany), and only high-quality RNA samples were used. Reverse transcription was performed using RevertAid Reverse Transcriptase (Thermo Fisher, USA). Both kits were used according to the manufacturer’s instructions. Real-time PCR was performed, and the relative expression levels of the virulence genes *vacA*, *cagA*, *ureA*, and *babA* were calculated using *16 s rRNA* as a housekeeping gene. Quantitative PCR reactions were performed using 12.5 μL of QuantiTect SYBR Green PCR Master Mix 2X (Qiagen, United States), 0.5 μL forward primer, 0.5 μL reverse primer (20 μM), 3 μL of cDNA, and RNase-free water to a final volume of 25 μL. The thermal cycling conditions were as follows: initial denaturation for 15 min at 95 °C, followed by 45 cycles of denaturation at 95 °C for 15 s, annealing at 55 °C for 30 s, and extension at 72 °C for 30 s. After that, a melting curve analysis was performed for one cycle as a finalization step, with a temperature range from 55 to 95 °C. Each primer pair produced a single, well-defined peak, confirming amplification specificity and the absence of primer-dimer formation (Supplementary Figure 1). The quantitative real-time PCR primer sequences are mentioned in Table 1. No-template controls (NTC) and no-reverse-transcription (no-RT) controls were included in all experiments and showed no detectable amplification, confirming the absence of contamination and genomic DNA interference.

**Table 1:** Primer sequences used for qRT-PCR.

Gene	Primer direction	Sequence 5’- 3’	Amplicon size (bp)	Reference
*cagA*	Forward Reverse	TGGCTAAAGCAACGGGTGAT CCGACTAGGGTTCCGTTCAC	172	Jin and Yang (2021)
*vacA*	Forward Reverse	CACTAACGCTGATGGCACGA GGACAGATTGACACCGCCTT	100	Jin and Yang (2021)
*babA*	Forward Reverse	TGCTCAGGGCAAGGGAATAA ATCGTGGTGGTTACGCTTTTG	116	Feng et al. (2024); Liu et al. (2024)
*ureA*	Forward Reverse	TCAAACCTTACCGCTGTCCC GCGACAGACCCGTTCAAATC	127	Liu et al. (2024)
*16 s rRNA*	Forward Reverse	CCCCACCTTCCTCCTCCTTA TCGTGTCGTGAGATGTTGGG	118	Liu et al. (2024)

The Stratagene MX3005P software was used to determine the amplification curves and threshold cycle (Ct) values for each reaction. To estimate the variation in gene expression in the RNA of the various samples, the Ct for each gene amplification was normalized to that of the *16S rRNA* gene amplified from the corresponding sample. All experiments were conducted in triplicates. Finally, the gene transcription fold changes were analyzed using the 2^−ΔΔCt^ method.

### 2.13 *In vivo* study

#### 2.13.1 Animals and experimental design

It is imperative to validate the functional and biological attributes of *L. plantarum* isolate L20 and *L. fermentum* isolate L22 through animal experiments to achieve practical applications.

Thirty-five adult male Sprague–Dawley rats (180–220 gm) were obtained from the Cairo Experimental Animals Farm, Egypt. All animal experiments were conducted in accordance with the ethical guidelines of the Institutional Animal Care and Use Committee (National Research Council Committee, 2011). The Ethics Committee for Experimental and Clinical Studies at the Faculty of Pharmacy, Cairo University (Egypt) approved our protocol (approval no. MI 3112).

**Figure 1:**
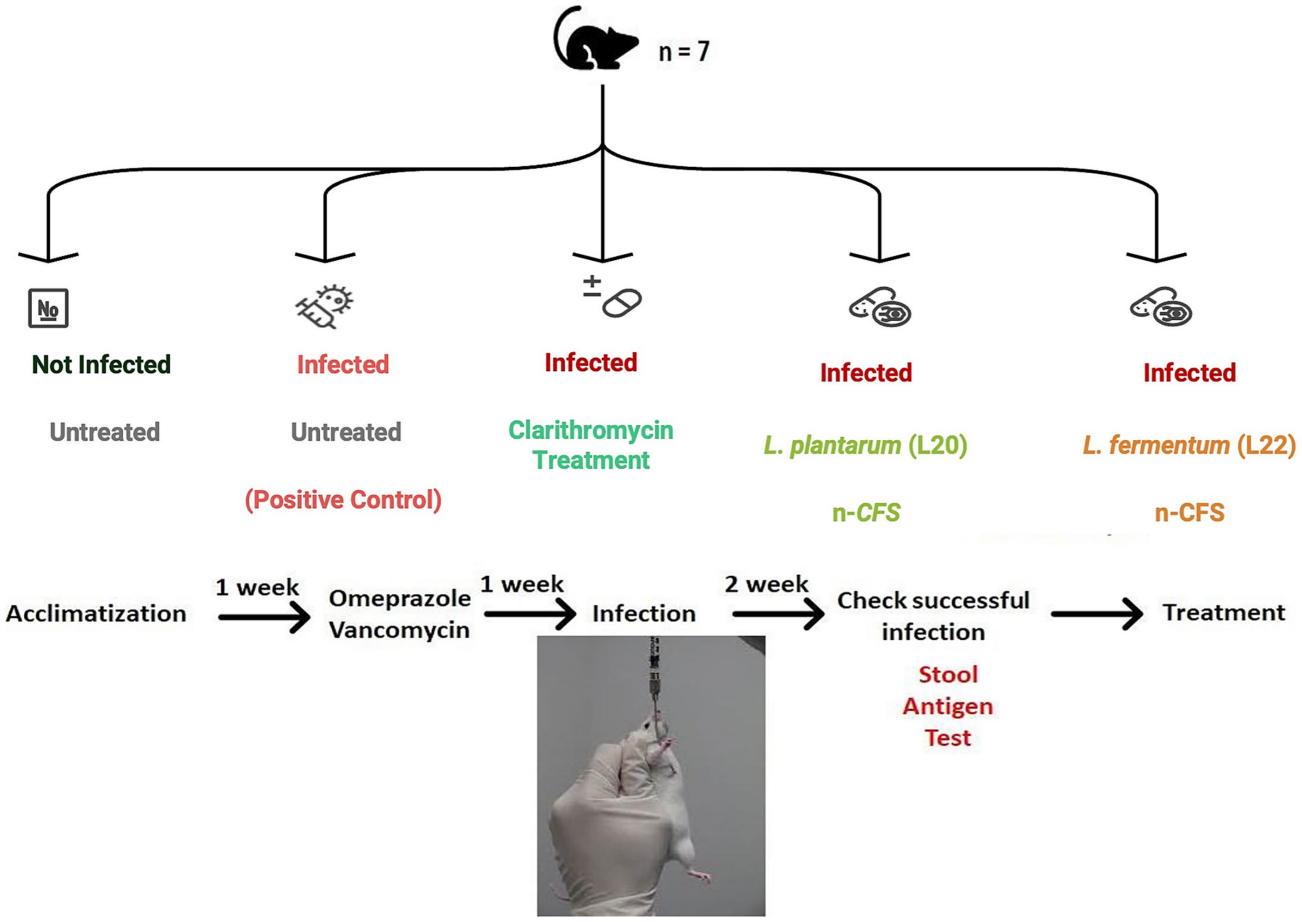
Schematic presentation of the in vivo experimental design for the administration of the selected isolates into the different animal groups.

After 1 week of acclimatization, the rats were randomly divided into five groups (*n* = 7 per group). Group 1 (G1): negative control (non-infected), Group 2 (G2): positive control (infected with *H. pylori*, untreated), Group 3 (G3): infected + clarithromycin treatment (standard treatment), Group 4 (G4): infected + *L. plantarum* isolate L20 n-CFS, Group 5 (G5): infected + *L. fermentum* isolate L22 n-CFS (Figure 1).

#### 2.13.2 Induction of *Helicobacter pylori* infection

Before *H. pylori* infection, the gastric microbial burden in rats was reduced by oral administration of omeprazole (25 mg/kg body weight) and vancomycin (25 mg/kg body weight) once daily for 7 days. Groups 2–5 were then infected with *H. pylori* (2 × 10^8^ CFU/mL) via oral gavage every other day for a total of five doses. Successful infection in the G2-G4 groups was confirmed by the end of the 3rd week post-infection using the HpSA test (GL-Scientific, Egypt) (Ibrahim et al., 2022).

#### 2.13.3 Treatment protocol

The n-CFSs from *L. plantarum* L20 and *L. fermentum* L22 were lyophilized using a freeze dryer (Martin Christ, Germany), distributed into small aliquots of equal weight, and stored at −20 °C. When needed, the lyophilized samples were reconstituted in sterile PBS to achieve the original volume before lyophilization (Lin and Pan, 2019).

Three weeks post-infection, the treatment regimens were initiated and administered for 21 consecutive days. Clarithromycin was administered to Group 3 30 mg/kg body weight once daily via oral gavage. Group 4 received *L. plantarum* L20 n-CFS (1 mL per rat via oral gavage once daily), while Group 5 received *L. fermentum* L22 n-CFS under the same conditions (1 mL, oral gavage, once daily). The n-CFS preparations were freshly prepared, neutralized to pH 7.0, and sterile-filtered before administration. Control Groups 1 and 2 received an equivalent volume of sterile phosphate-buffered saline (PBS) via oral gavage following the same schedule. After 24 h from the last treatment dose, all animals were euthanized using an intraperitoneal lethal dose of pentobarbital sodium (200 mg/kg) (Bayne, 1996). The stomachs were then aseptically removed, weighed, bisected, and homogenized in sterile PBS using a tissue homogenizer (Germany) at 1500 rpm for 20 s (Li et al., 1998).

The gastric homogenates were serially diluted in PBS. From each dilution, 20 μL was spotted on Columbia blood agar containing DENT supplement and incubated under microaerophilic conditions at 37 °C for 72 h. Colonies with characteristic *H. pylori* morphology (small, gray, translucent, non-hemolytic, 1–2 mm in diameter) were confirmed by urease testing through inoculation onto *ureA* agar slants (LabM, United Kingdom), where a characteristic color change from yellow to pink within 24 h confirmed urease positivity, consistent with *H. pylori* identification were counted and expressed as colony-forming units per gram of stomach tissue (CFU/g) (Kadkhodaei et al., 2020).

### 2.14 Proteomic analysis

The proteomic approach was implemented to identify the *L. plantarum* L20 n-CFS proteins that were involved in its activity.

#### 2.14.1 Protein extraction

Protein extraction from *L. plantarum* L20 supernatants was done according to the method outlined by Saadeldin et al. (2020), with some modifications. The proteins were precipitated by adding acetone to the sample four times. The proteins were centrifuged for 30 min at 15,000 rpm and 4 °C. The protein pellet was reconstituted in 200 μL of 8 M urea lysis buffer. The samples underwent centrifugation at 4 °C and 10,000 rpm for 30 min. Ultimately, the total protein extracted was quantified using the Bradford assay deNovix DS-11 FX Series (Siragusa et al., 2011). The measurements were conducted at 595 nm before digestion.

#### 2.14.2 Enzymatic digestion of protein

A total of 30 μg of protein sample was reduced and alkylated for 45 min with 200 mM dithiothreitol (DTT) (Merck, Germany) and 1 M iodoacetamide (IAA) (Sigma, United States), respectively. To facilitate protein digestion, 1 μg/μL of porcine trypsin (Sigma, Germany) was added and sonicated. The digested peptide solution was acidified with 100% formic acid (FA) to a final pH of 2.0. The obtained peptides were further quantified using a bicinchoninic acid (BCA) assay kit, following the manufacturer’s instructions (Wiśniewski and Gaugaz, 2015).

#### 2.14.3 LC MS/MS analysis

The nano-LC MS/MS analysis was performed using an Eksigent nanoLC 400 autosampler coupled to an Ekspert nanoLC 425 pump and a TripleTOF 5,600 + mass spectrometer (SCIEX, Canada). The peptide solution was injected and trapped onto 5 μm ChromXP C18-CL (SCIEX, Germany) at a flow rate of 10 μL/min for 5 min using a mobile phase containing 0.1% FA. Peptides were then eluted using a 3 μm, ChromXP C18-CL reverse-phase column 120 A, 150 × 0.3 mm (SCIEX, Germany), using a linear gradient 3–80% solution of mobile phase acetonitrile (ACN) containing 0.1% formic acid (FA) with a flow rate of 5 μL/min for 55 min. In positive mode, the set ranges for MS and MS/MS were 400–1,250 m/z and 170–1,500 m/z, respectively. The 40 most abundant ions were successively chosen in data-dependent acquisition (DDA) mode with charge states 2–5. Before and within the sample batches, calibration was performed to correct any possible TOF deviation to ensure accurate analysis. The MS proteomics data have been deposited to the ProteomeXchange Consortium via the PRIDE (Perez-Riverol et al., 2025) partner repository with the dataset identifier PXD064835.

#### 2.14.4 Proteomics data analysis

The raw files were converted to Mascot generic format (mgf) using AB SCIEX MS Data. The MS/MS spectra were analyzed using Protein Pilot software (version 5.0.1.0, 4,895) and the Paragon Algorithm (version 5.0.1.0, 4,874) against the Uniprot *Lactiplantibacillus plantarum* (Swiss-Prot and TrEMBL database containing 11,544 proteins). Parent proteins were identified using the highest score for a given peptide mass, which corresponded to the most accurate prediction in the database.

The following parameters were used in the database searches: trypsin as a proteolytic enzyme with a maximum of two missed cleavages, oxidation of methionine, and protein N-terminal acetylation as variable modifications and carbamidomethyl for cysteine as a fixed modification. The peptide-spectrum match (PSM) was then filtered based on high confidence and Mascot search engine rank 1 in peptide identification to ensure that the overall false discovery rate remained below 0.01. Proteins exhibiting a single peptide hit were retained. The functions of the identified proteins were annotated using Gene Ontology (GO) analysis, and the Kyoto Encyclopedia of Genes and Genomes (KEGG) database was used to analyze the metabolic pathways.

### 2.15 Statistical analysis

GraphPad Prism software version 9.0 was used to perform statistical analyses. All results from the three independent experiments are presented as mean ± standard deviation (SD). The differences between the mean values of the control and tested groups were evaluated using one-way ANOVA, followed by Tukey’s post-hoc test. Student’s t-test was used for unpaired samples. Multiple comparisons between various groups were analyzed using two-way ANOVA. Values at *p* < 0.05 were regarded as statistically significant.

## 3 Results

### 3.1 Isolation and preliminary identification of lactic acid bacteria

A total of 119 samples were collected from different dairy products, including milk, yogurt, and cheese. All recovered isolates were Gram-positive cocci or rod-shaped, catalase- and oxidase-negative. Furthermore, all isolates were able to grow optimally in the presence of 3 and 4.5% NaCl (w/v). In addition, all isolates exhibited *γ*-hemolytic activity on blood agar plates. All isolates were presumptively identified as LAB and subjected to further examination.

### 3.2 Bile salt tolerance

Upon exposure to 0.3% bile salt for 4 h, all LAB isolates exhibited a high level of tolerance. Generally, the bile tolerance of nearly all LAB isolates exceeded 50%. Among them, fifty isolates, representing 42% of the total isolates, were the most resistant to bile salt conditions, with growth inhibition ranging from 1.36 ± 0.27% to 11.95 ± 0.52%.

### 3.3 Survival of lactic acid bacteria isolates under gastric conditions

Colonization of the gastrointestinal tract by LAB isolates was conducted by assessing their survival at low pH in simulated gastric environments. Most isolates exhibited tolerance levels exceeding 40%. Among them, 37 isolates exhibited the highest survival values, ranging from 80.01 ± 0.84% to 89.61 ± 2.8%.

### 3.4 Auto-aggregation and co-aggregation examination

Our findings indicated that the aggregation and co-aggregation capabilities of the tested isolates exhibited a time-dependent manner, with a peak observed at 24 h. All bacterial isolates displayed a certain degree of auto-aggregation after 1 h, ranging from 0.97 to 12.74%. Auto-aggregation progressively increased over time, reaching peak values between 22.64 ± 1.93% and 70.26 ± 2.47%.

Similarly, all the LAB isolates demonstrated the ability to co-aggregate with *H. pylori in vitro*. Co-aggregation ranged from 0.31 ± 0.44% to 17.24 ± 0.94% after 1 h, and reached their highest levels after 24 h, ranging from 11.61 ± 0.69% to 58.68 ± 0.61%. Notably, 19 LAB isolates displayed high co-aggregation capacity, with values ≥ 50%. Specifically, L20 showed 50.37 ± 0.53%, L22 showed 58.68 ± 0.61%, and L40 showed 52.43 ± 1.79% co-aggregation with *H. pylori*. The top-performing isolates were selected for further investigations because they demonstrated acid survival ≥ 80%, bile tolerance ≥ 85–90%, auto-aggregation ≥ 60%, and co-aggregation ≥ 50%.

### 3.5 Antibiotic susceptibility testing

The 19 selected LAB isolates were evaluated for their antibiotic susceptibility by the Kirby-Bauer disc method, based on the protocol outlined by CLSI (2020). All isolates showed variable susceptibility to antibiotics. All LAB isolates exhibited resistance to cefotaxime, kanamycin, and streptomycin, except for L20, L22, and L40, which were the most sensitive. Nonetheless, all isolates were sensitive to penicillin, ampicillin, chloramphenicol, and tetracycline (Supplementary Table 1).

### 3.6 Screening of the antimicrobial activity of lactic acid bacteria

The antimicrobial activities of the CFSs of the 19 selected LAB isolates against *H. pylori* were evaluated using the agar diffusion method. Among them, 16 isolates exhibited antimicrobial activity against *H. pylori*. However, after neutralization to pH 6.5 (n-CFS), almost all isolates lost their inhibitory activities.

### 3.7 Effect of lactic acid bacteria supernatants on urease inhibition of *Helicobacter pylori*

**Figure 2:**
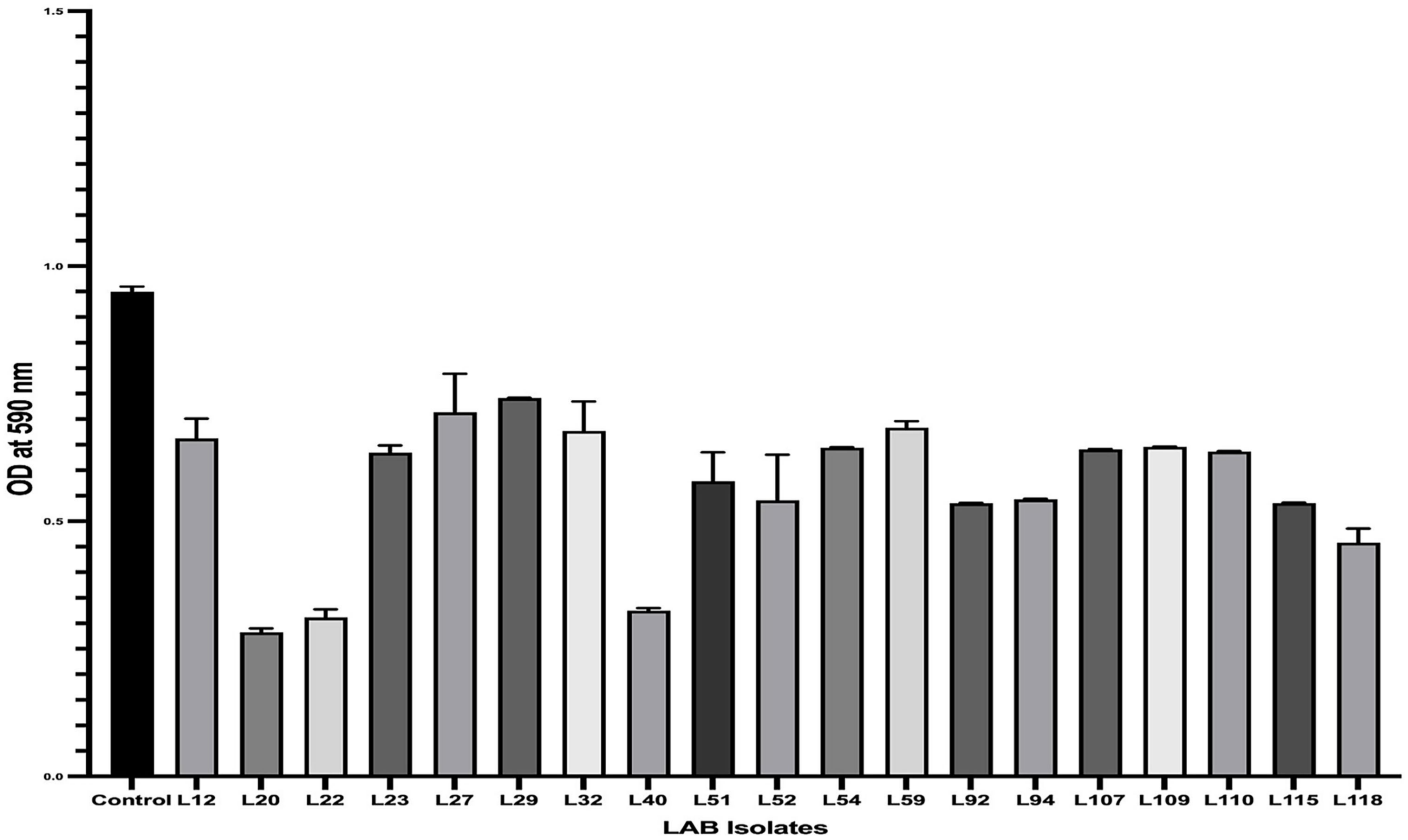
The effect of neutralized cell-free supernatants of LAB isolates on *H. pylori* urease activity. The urease activity of *H. pylori* alone (control) and *H. pylori* treated with n-CFS of LAB isolates was quantified by urease assay and measured at an optical density of 590 nm. Data are presented as the mean ± SD. Statistical difference was determined by one-way ANOVA test and Tukey’s *post hoc* test with a significance level at *p* < 0.05. The charts were generated using GraphPad Prism 9 software.

The urease inhibition ability of the 19 selected LAB isolates was evaluated by quantifying the reduction in pH within a urea-containing medium. All tested LAB isolates exhibited different degrees of urease inhibition, ranging from 21.14 ± 0.1% to 69.95 ± 1.14% (Figure 2). Among the isolates, L20 demonstrated the highest urease inhibition (69.95 ± 1.14%), followed by L40 (67.03 ± 1.51%) and L22 (66.34 ± 2.03%). Moderate inhibition levels were observed in isolates such as L52 (49.48 ± 2.26%), L115 (42.93 ± 0.53%), L92 (42.56 ± 1.51%), and L94 (42.03 ± 0.76%). In contrast, lower inhibitory effects were recorded for isolates L27 (20.17 ± 6.09%) and L29 (21.14 ± 0.10%).

### 3.8 Effect of lactic acid bacteria supernatants on biofilm formation of *Helicobacter pylori*

**Figure 3:**
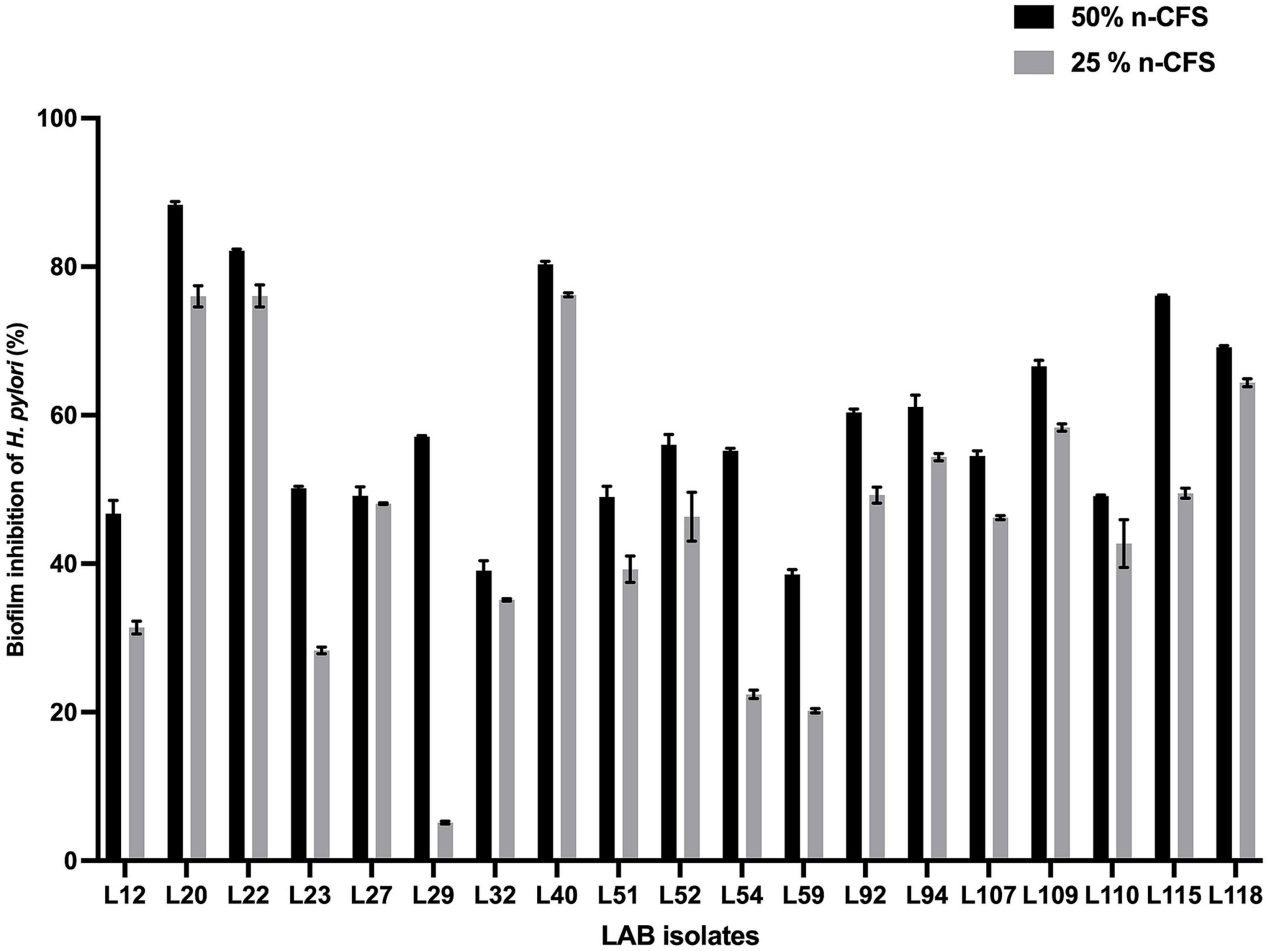
Percentage of biofilm inhibition following the treatment with 50 and 25% of n-CFS of 19 selected LAB isolates. The data are demonstrated as the mean ± SD of triplicate values that were obtained from independent experiments. Statistical difference was determined by one-way ANOVA test and Tukey’s post hoc test with a significance level at *p* < 0.05. The charts were generated using GraphPad Prism 9 software. n-CFS is the cell-free supernatant neutralized to pH 6.5.

All the tested isolates exhibited varying degrees of biofilm inhibition (Figure 3). At 50% n-CFS, the highest biofilm inhibition was observed for isolates L20 (88.32 ± 0.46%), L22 (82.15 ± 0.22%), and L40 (80.30 ± 0.43%), followed by L115 (76.09 ± 0.13%) and L118 (69.15 ± 0.22%). Moderate inhibition was recorded in isolates such as L109 (66.57 ± 0.81%), L94 (61.13 ± 1.60%), and L92 (60.35 ± 0.50%). In contrast, relatively lower inhibition was observed in isolates L32 (39.05 ± 1.35%) and L59 (38.54 ± 0.66%). At 25% n-CFS, a general decline in biofilm inhibition was evident, indicating a concentration-dependent effect. However, certain isolates maintained strong inhibitory activity even at the lower concentration, particularly L22 (76.05 ± 1.49%), L40 (76.20 ± 0.29%), and L20 (74.95 ± 0.36%). Notably, L118 (64.38 ± 0.54%) and L109 (58.35 ± 0.50%) also retained considerable activity.

### 3.9 Molecular identification by 16S rRNA sequencing

Among all the tested isolates, three potential isolates (L20, L22, and L40) exhibited the most balanced and superior probiotic properties. Specifically, L20 and L22 showed the highest bile tolerance (97.48 and 98.11%, respectively), strong acid survival (>84%), and substantial co-aggregation with *H. pylori* (≥50%). Moreover, L40 demonstrated the highest auto-aggregation capacity (70.26%) among all tested isolates. In addition, these three strains exhibited pronounced biofilm inhibition (≥78%) and urease inhibition (>66%). Accordingly, these isolates were selected as candidates for molecular identification using 16S rRNA gene sequencing.

**Figure 4:**
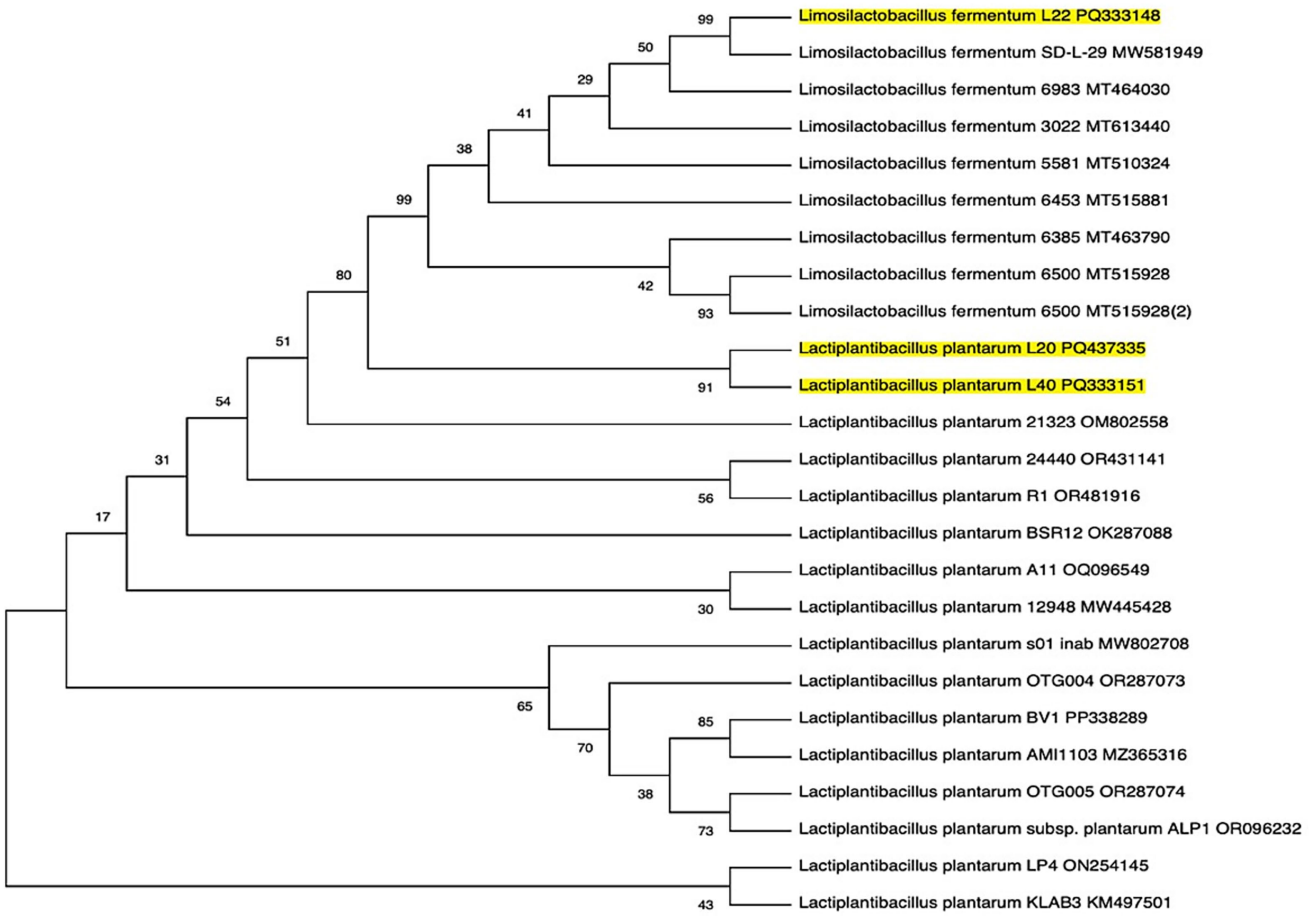
Comparative phylogenetic analysis of *L. plantarum* L20, L40, and *L. fermentum* L22 with the other 22 strains was conducted using MEGAX. Evolutionary distances were generated using the neighbor-joining method. The bootstrap consensus tree was inferred from 1,000 replicates.

Isolates L20 and L40 were identified as *Lactiplantibacillus plantarum* (accession no. PQ437335 and PQ333151), exhibited 100 and 99.8% identity, respectively. In contrast, isolate L22 was identified as *Limosilactobacillus fermentum* (accession no. PQ333148) with 98.17% identity. Additionally, the three sequences were assembled for phylogenetic analysis and aligned with another 22 closely related species (Figure 4).

### 3.10 Growth curve analysis

The growth curve was conducted to determine the appropriate dilutions of n-CFS of *L. plantarum*20, *L. fermentum*22, and *L. plantarum*40, which allowed the growth of *H. pylori* over a duration of 24 h. The n-CFS at dilutions of 50% exhibited no effect against *H. pylori* compared to the control group. However, partial inhibition was still observed with undiluted supernatants, indicating that the 50% dilution could serve as a suitable candidate for further experiments (Supplementary Figure 2).

### 3.11 Effect of *Lactiplantibacillus plantarum* and *Lactiplantibacillus fermentum* supernatants on gene expression levels

All the virulence genes of *H. pylori* (*vacA*, *cagA*, *ureA*, and *babA*) were significantly downregulated upon treatment with 50% n-CFS of *L. plantarum* L20, *L. fermentum* L22, and *L. plantarum* L40. Specifically, *vacA* mRNA levels decreased by 79 ± 0.03%, 46 ± 0.04%, and 40 ± 0.07%, respectively. Similarly, *cagA* mRNA levels exhibited fold decreases of 68 ± 0.03%, 52 ± 0.05%, and 38 ± 0.05%, respectively. For *ureA* mRNA, the observed reductions were 90 ± 0.02%, 69 ± 0.03%, and 54 ± 0.04%, respectively. Notably, the most substantial reductions were observed in *babA* mRNA levels, with 96 ± 0.01%, 87 ± 0.01%, and 70 ± 0.03%, respectively, under the same treatment conditions.

**Figure 5:**
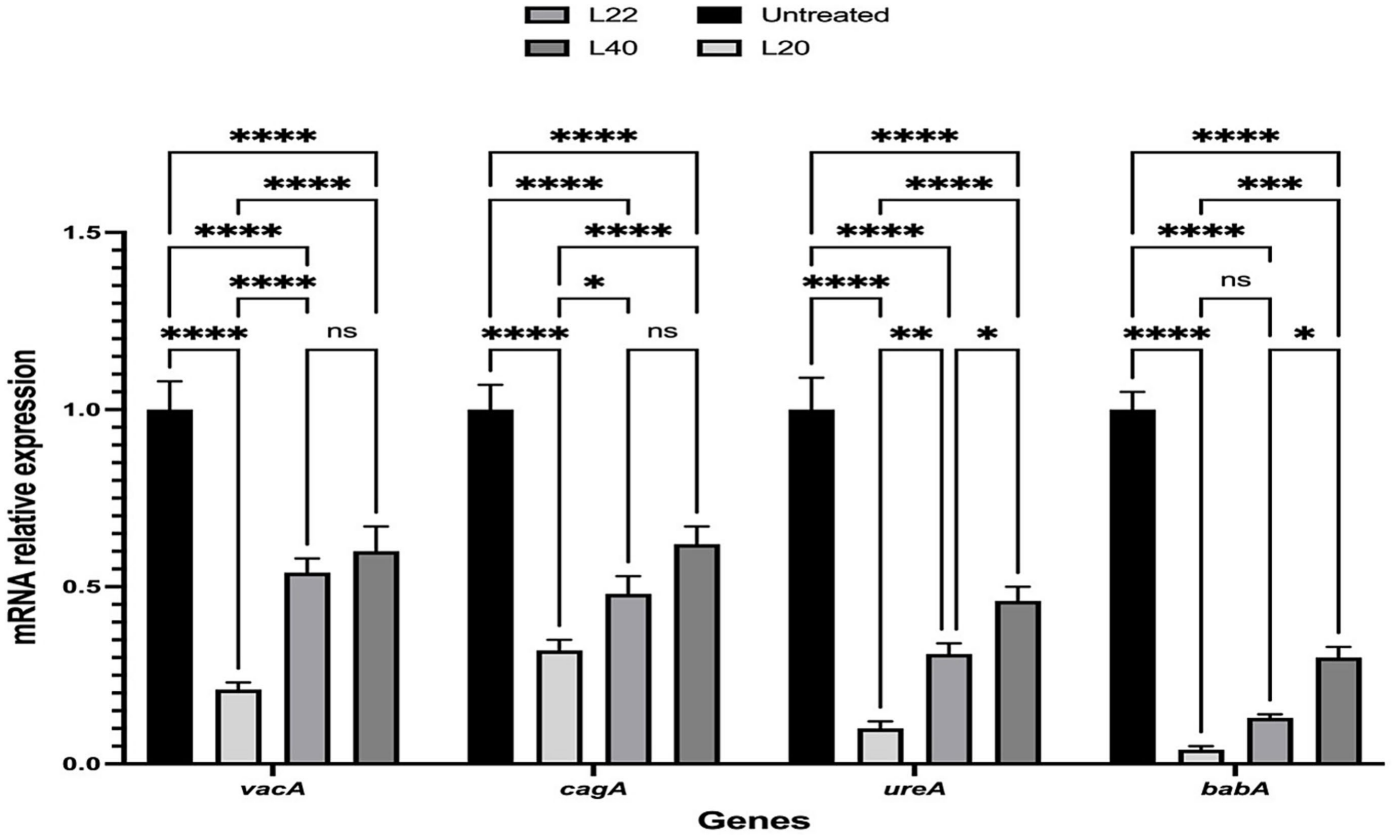
Fold changes in *vacA*, *cagA*, *ureA*, and *babA* gene expression of *H. pylori* transcription factors analyzed by qRT-PCR after treatment with different groups (*L. plantarum* 20, *L. fermentum* 22, and *L. plantarum* 40 neutralized CFS) relative to the untreated group (without CFS). Target mRNA levels were normalized to those of the housekeeping gene, 16 s rRNA. The differences between groups were examined using two-way analysis of variance (ANOVA) followed by Tukey’s test. Error bars represent the standard deviation. ns, non-significant difference;* = *p* < 0.05; ** = *p* < 0.01; *** = *p* < 0.001; **** = *p* < 0.0001: significant relative to the negative control (*H. pylori* alone). The charts were generated using GraphPad Prism 9 software.

Among the treated groups, *L. plantarum* L20 showed the highest downregulation effect on the *vacA*, *cagA*, and *ureA*, whereas *L. plantarum* L40 and *L. fermentum* L22 showed no significant difference between them. In addition, a significant downregulation of *babA* expression was observed in all experimental groups, whereas the groups *L. plantarum* L20 and *L. fermentum* L22 were not significantly different compared with the untreated group (Figure 5).

### 3.12 Effect of *Lactiplantibacillus plantarum* 20 and *Lactiplantibacillus fermentum* 22 supernatants on *Helicobacter pylori* colonization

The therapeutic efficacy of *L. plantarum* L20 and *L. fermentum* L22 n-CFSs against *H. pylori* infection was evaluated using a Sprague–Dawley rat model. Following the 21-day treatment, recovered colonies were identified as *H. pylori* based on their characteristic morphology on DENT-supplemented selective media under microaerophilic conditions, which was further supported by urease positivity. Quantitative analysis of *H. pylori* colonization was subsequently performed by serial dilution and colony counting of the gastric homogenates.

**Figure 6:**
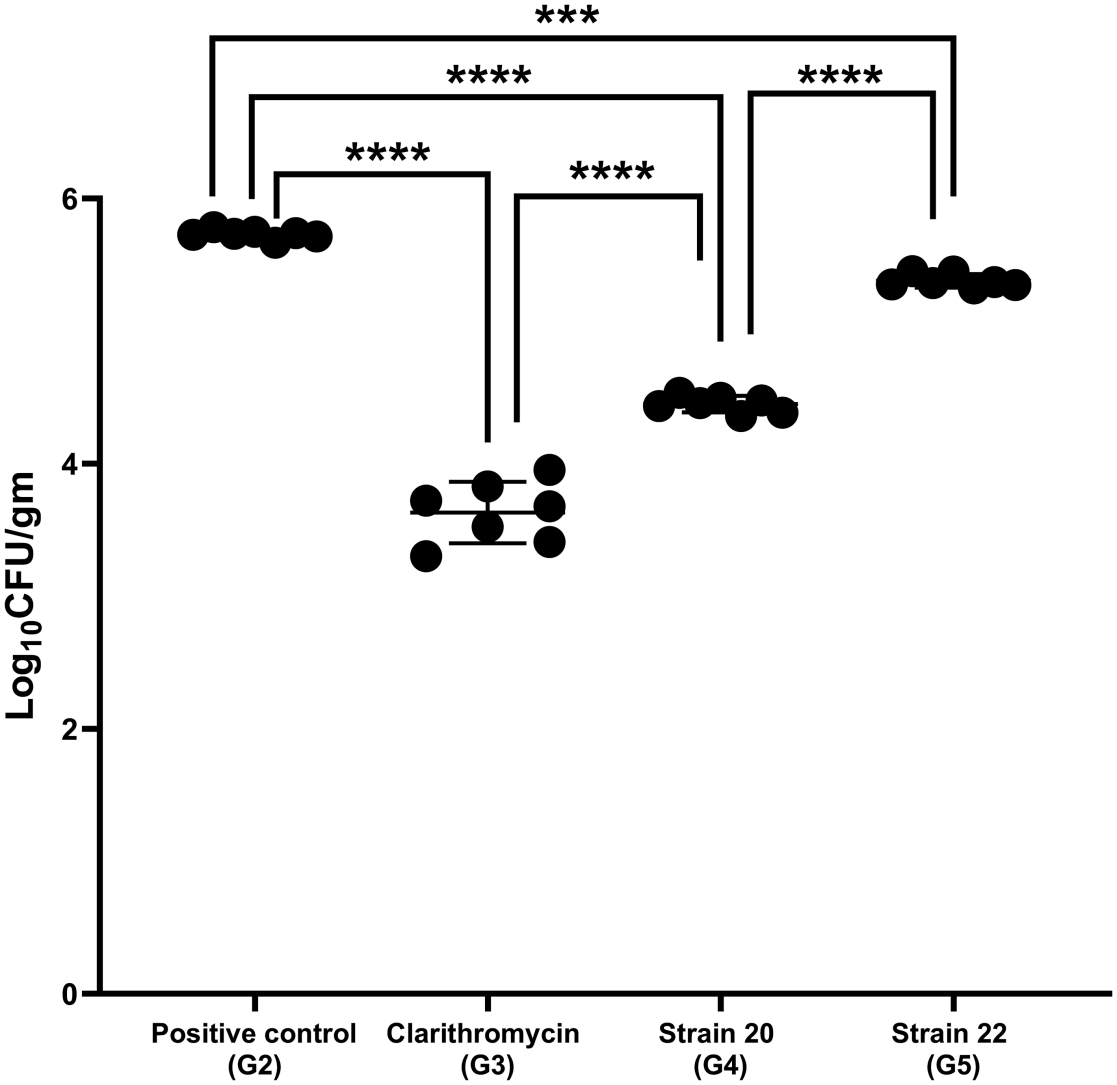
Effect of clarithromycin and experimental strains on *H. pylori* colonization in vivo. The graph shows the bacterial load measured as log₁₀ CFU/g of gastric tissue in different treatment groups. The positive control group (G2) included rats with untreated *H. pylori* infection. The treatment groups included those treated with clarithromycin (G3), n-CFS of *L. plantarum* L20 (G4), and n-CFS of *L. fermentum* L22 (G5). Each dot represents an individual animal, with horizontal lines indicating the mean ± standard error. Statistical significance between the indicated groups is denoted by asterisks (****p < 0.0001). Clarithromycin treatment resulted in the most significant reduction in bacterial load, followed by *L. plantarum* L20, whereas *L. fermentum* L22 showed a more modest reduction relative to the positive control.

The negative control group (G1, uninfected) showed no detectable *H. pylori* colonies. The positive control group (G2, *H. pylori*-infected, untreated) showed substantial colonization, corresponding to approximately (5 ± 0.44) × 10^5^ CFU/g of stomach tissue. Treatment with the standard antibiotic clarithromycin (G3) significantly reduced *H. pylori* colonization, with (4.8 ± 2.47) × 10^3^ CFU/g, representing a 2.1 log reduction relative to the untreated infected group (*p* < 0.001). Treatment with n-CFS of *L. plantarum* L20 (G4) resulted in a 1.28 log reduction, whereas n-CFS of *L. fermentum* L22 (G5) showed a more modest but still significant 0.35 log reduction compared to the untreated infected group (Figure 6).

### 3.13 Proteomic analysis

#### 3.13.1 proteome profile of *Lactiplantibacillus plantarum* L20 cell-free supernatants

The peptide sequences were mapped against the SwissProt protein database, resulting in the identification of 27 proteins. Based on the highest conf (peptide) value of *L. plantarum* proteins, phytase, acetolactate synthase, glycoside hydrolase, NADP-specific glutamate dehydrogenase, and glycosyltransferase were the most abundant proteins. Approximately 18% of the identified proteins were classified as hypothetical proteins.

#### 3.13.2 Gene ontology and Kyoto encyclopedia classification of proteins in CFS of *Lactiplantibacillus plantarum* L20

**Figure 7:**
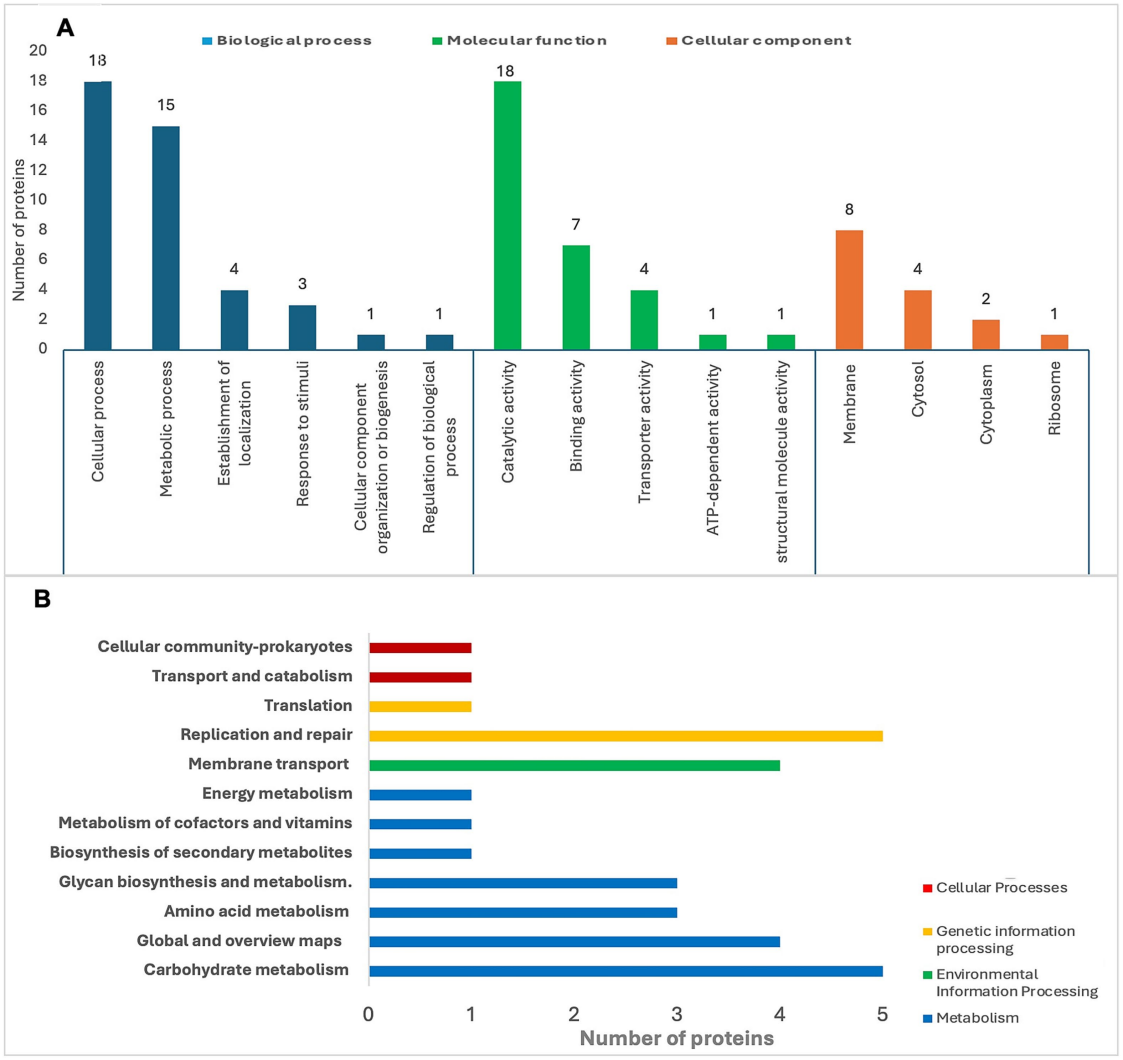
Proteomic analysis of identified proteins in *L. plantarum* L20. **(A)** Gene Ontology (GO) annotation. The GO categories included biological processes, cellular components, and molecular functions. **(B)** Kyoto Encyclopedia of Genes and Genomes (KEGG) classification pathways. The KEGG pathways of the four branches included metabolism, genetic information processing, environmental information processing, and cellular processes. The number of proteins in each category is indicated by each digit above the bar.

According to the annotation analysis of UniProtKB and Gene Ontology databases, differentially expressed proteins play essential roles in biological processes, cellular components, and molecular functions (Figure 7A). In the present study, 22 of the 27 proteins in the n-CFS derived from *L. plantarum* were identified in the molecular function category in the GO database. The main molecular function of these proteins included catalytic activity (66.7%), followed by binding activity, transporter activity, ATP-dependent activity, and structural molecule activity. In contrast, approximately 19 proteins were annotated for biological processes, including gluconeogenesis, glycolytic process, amino acid biosynthesis, transmembrane transport, cell wall organization, cell division, and translation (Supplementary Table 2).

Moreover, the functional annotation of KEGG revealed that the differentially expressed proteins were notably focused on carbohydrate metabolism, biosynthesis of secondary metabolites, membrane transport, amino acid metabolism, and glycan biosynthesis (Figure 7B). Among these, there were pathways associated with the prokaryotic defense system, such as the exonuclease SbcCD subunit D, transcription-repair-coupling factor, and type I restriction-modification system specificity subunit S.

## 4 Discussion

The prevalence of *H. pylori* infection has raised considerable concerns worldwide (Baj et al., 2021). In addition, *H. pylori* prevalence in Egypt is notably higher than the global average, with considerable antimicrobial resistance (Alsulaimany et al., 2020). Hence, there is a growing need for alternative therapeutic approaches to overcome antibiotic-resistant strains and improve their eradication rate. One promising avenue of research is the use of lactic acid bacteria, which are widely recognized for their potential antimicrobial properties (Ruggiero, 2014).

A total of 119 LAB isolates recovered from different dairy products were Gram- positive, cocci or rod-shaped, catalase- and oxidase-negative, which were preliminarily identified as LAB. Our primary focus was directed toward the isolation of *Lactobacillus* species, which are considered pivotal potential probiotic isolates (Todorov et al., 2016). However, there was considerable variation in the morphological characteristics of the isolates, with bacilli being the dominant cell type, which is consistent with other studies (Bazireh et al., 2020).

Furthermore, all LAB isolates exhibited exceptional tolerance to 0.3% bile salt, with survival rates exceeding 50% after 4 h of exposure. Similarly, most isolates exhibited tolerance to artificial gastric juice, with survival rates exceeding 40%. Based on our results, the survival rate under gastric conditions varied notably between the strains, suggesting a strain-specific pattern. These disparities in resistance may be attributable to the expression of resistance-associated proteins in LAB isolates (Hamon et al., 2011; Butorac et al., 2020). In addition, these tolerance capabilities can be attributed to the bilayer membrane structure, which enables straightforward tolerance to adverse conditions (Khushboo et al., 2023).

Our findings revealed that all LAB isolates in this study exhibited a certain degree of auto-aggregation and co-aggregation from the initial time of 1 h, reaching a peak at 24 h. A time-dependent increase in auto-aggregation and co-aggregation abilities was observed, which is consistent with other studies (Li et al., 2020; Zhou et al., 2021).

Moreover, 19 LAB isolates showed a high potential for co-aggregation (exceeding 50%). From these, the three selected candidates, *L. plantarum* L20, *L. fermentum* L22, and *L. plantarum* L40, were identified as the top-performing isolates. This high co-aggregation capacity suggests a strong potential for direct interaction with *H. pylori*, which may contribute to competitive exclusion and interference with pathogen adhesion. These interactions could enhance persistence within the gastric environment and support the maintenance of local microbial balance, thereby limiting pathogen colonization (Wang et al., 2024).

Susceptibility to antibiotics and lack of transferable antibiotic-resistance genes are important criteria for bacterial safety (Campana et al., 2017). The current study indicated that no antibiotic resistance patterns were observed for penicillin, ampicillin, chloramphenicol, and tetracycline. However, most of the tested strains were resistant to cefotaxime, kanamycin, and streptomycin, except for L20, L22, and L40 strains. The absence of resistance to these commonly used antibiotics is highly favorable for the potential probiotic application of these LAB strains (Anisimova et al., 2022). This suggests a low risk of contributing to the spread of resistance to common broad-spectrum antibiotics. Certain LAB frequently exhibit resistance to streptomycin, kanamycin, and cefotaxime, which is indicative of intrinsic resistance (Mir et al., 2018; Campedelli et al., 2019).

Our results showed that most LAB isolates exhibited antimicrobial activity against *H. pylori*. Furthermore, after neutralization of CFS, all the LAB isolates completely lost their antibacterial activity. The obtained data obviously established the significant role of organic acids in the antimicrobial activity of the tested isolates (Somashekaraiah et al., 2019). Based on these findings, our LAB strains are highly promising probiotics with the inherent capacity to survive in the gastrointestinal tract, colonize the gut effectively, and actively combat pathogenic microorganisms. Owing to their numerous capabilities, these LAB strains were potential candidates for further investigations.

Urease inhibition assays further revealed that LAB-derived n-CFS significantly suppressed urease activity, a crucial virulence factor that enables *H. pylori* to survive in the acidic gastric environment (Shaalan et al., 2024). The results of our study indicated that the n-CFS of 19 isolates exhibited substantially higher urease inhibition. Notably, isolates such as L20, L22, and L40 exhibited the highest urease inhibitory activity. Urease inhibition following neutralization confirms that this effect is not solely due to acidic pH (Szczerbiec et al., 2024) but also involves pH-independent bioactive compounds (Ismael et al., 2022).

Similarly, strong anti-biofilm activity was observed among the tested isolates. At higher concentrations (50% n-CFS), isolates L20, L22, and L40 showed the greatest inhibition of *H. pylori* biofilm formation, with inhibition levels exceeding 75%. This antibiofilm activity may be attributed to the diverse bioactive components released by LAB into the extracellular CFS, such as bacteriocins, biosurfactants, exopolysaccharides, and enzymes (Hu et al., 2021; Molham et al., 2021), which may hinder biofilm formation. Nevertheless, n-CFS from selected LAB isolates exhibited no antibacterial activity, indicating that the biofilm inhibition observed in this study was not attributable to antimicrobial components, such as organic acids or bacteriocins (Molham et al., 2021).

Molecular identification using 16S rRNA sequencing confirmed that the most promising isolates L20 and L40 belonged to *Lactiplantibacillus plantarum*, while L22 was identified as *Limosilactobacillus fermentum*. These candidates are well-recognized for their superior probiotic properties, and their consistent performance across multiple assays highlights their multifunctional potential with both antimicrobial and anti-virulence capabilities, supporting their prioritization for further evaluation (Bazireh et al., 2020).

Additionally, a significant downregulation of key *H. pylori* virulence genes has been observed following exposure to *L. plantarum* L20, *L. fermentum* L22, and *L. plantarum* L40. These findings suggest that these strains not only inhibit bacterial growth but also attenuate pathogenicity at the transcriptional level. This was evidenced by the inhibition of urease activity through decreased *ureA* expression, reduced bacterial adhesion through downregulation of *babA*, and, importantly, mitigation of cancer risk through decreased expression of major virulence factors such as *vacA* and *cagA*. Among the tested isolates, L20 (*L. plantarum*) demonstrated the most pronounced effect, achieving reductions of 78%, 69%, 90%, and 96%, respectively. The marked suppression of *ureA* expression is consistent with its strong urease inhibitory activity observed phenotypically. The observed combined effects suggest possible synergistic interactions among the tested isolates in suppressing *H. pylori* pathogenicity. These results highlight the potential of *Lactiplantibacillus plantarum* L20 n-CFS as an innovative strategy for *H. pylori* infection.

In order to achieve practical applications, it is imperative to validate the functional and biological attributes of *L. plantarum* L20 and *L. fermentum* L22 through animal experiments. In the *in vivo* model, clarithromycin achieved a greater reduction in *H. pylori* colonization (2.1-log) compared with *L. plantarum* L20 n-CFS (1.28-log) and *L. fermentum* L22 n-CFS (0.35-log). Although L20 n-CFS produced a statistically significant decrease in bacterial load relative to the untreated control (*p* < 0.0001), its effect remained lower than that of antibiotic therapy, confirming that clarithromycin remains the gold standard for *H. pylori* eradication. These findings suggest that L20 n-CFS may contribute to partial suppression of bacterial colonization, potentially through anti-virulence or growth-modulating mechanisms rather than direct bactericidal activity. In this context, its role may be better positioned as an adjunctive strategy alongside conventional antibiotic therapy, particularly in light of increasing clarithromycin resistance (Molina-Infante and Gisbert, 2014). However, further studies are required to optimize the dosing, evaluate the combination regimens, and confirm the clinical relevance.

The modest reduction observed with L22 n-CFS, despite statistical significance (*p* = 0.0001), suggests a limited biological impact under the tested conditions and is unlikely to support a standalone therapeutic application. Its potential application may be better explored in combination regimens with antibiotics or other probiotic strains.

An important observation emerging from this study is the pronounced strain specificity of anti-*H. pylori* activity. Despite both *L. plantarum* L20 and *L. fermentum* L22 being LAB isolates with strong probiotic properties *in vitro*, they demonstrated markedly different efficacy *in vivo*. This finding underscores that LAB-derived n-CFS activity against *H. pylori* cannot be generalized across species or strains. Future studies should systematically compare diverse LAB collections to identify the component responsible for anti-*H. pylori* efficacy, strengthening the basis for selecting the most effective strain-specific candidates.

To elucidate the potential mechanisms underlying the anti-*H. pylori* activity of *L. plantarum* L20, a proteomic analysis of its neutralized n-CFS was performed. Proteomic analysis identified 27 proteins mainly involved in metabolism, transport, and biosynthesis. The most abundant proteins included phytase, acetolactate synthase, and glucose-6-phosphate isomerase, suggesting a multifactorial anti-*H. pylori* mechanism involving metabolic interference, environmental modulation, and inhibition of virulence-associated processes. For instance, phytase may disrupt biofilm integrity through the degradation of structural polysaccharides (Priyodip et al., 2017), while acetolactate synthase may contribute to organic acid production and create unfavorable conditions for *H. pylori* survival (Kiousi et al., 2023). In addition, enzymes such as glycoside hydrolase, glucose-6-phosphate isomerase, and NADP-specific glutamate dehydrogenase have a major impact on numerous biological processes, including carbohydrate metabolism and energy production, potentially supporting the synthesis of antimicrobial metabolites (Huidrom et al., 2024). Glycosyltransferases, which are essential for glycan and exopolysaccharide biosynthesis (Wardman and Withers, 2024), may enhance adhesion and form a protective barrier (Mazzeo et al., 2025). This finding aligns with the observed strong adhesion and co-aggregation properties of *L. plantarum* L20. Additionally, transport systems, including ABC transporters and PTS components, support the active secretion and uptake of metabolites, which may facilitate the delivery of bioactive compounds responsible for antimicrobial and anti-virulence effects (Bilsing et al., 2023).

Importantly, although some identified proteins are associated with pathways that may resemble virulence-related systems in pathogenic bacteria, in the context of probiotic organisms, these functions are primarily linked to environmental adaptation, metabolic efficiency, and gastrointestinal survival. This distinction underscores the safety profile of *L. plantarum* L20 and supports its potential application as a functional probiotic.

This study provides preliminary evidence supporting the potential of *L. plantarum* L20 neutralized-CFS as a complementary strategy for managing *H. pylori* infection. However, the molecular mechanisms underlying the observed antimicrobial and anti-virulence effects remain unclear and require further investigation using fractionation, enzymatic treatments, and analysis of purified bioactive components.

Despite these promising outcomes, several limitations should be considered. Histological evaluation of gastric mucosal tissue was not performed in this study because the *in vivo* experiment was specifically designed to assess the effect of n-CFS on *H. pylori* colonization. Future studies will be required to comprehensively evaluate the therapeutic impact of n-CFS on gastric tissue pathology. In addition, the study was conducted using a single reference strain (*H. pylori* ATCC 43504), which limits the generalizability of the findings given the known genetic and phenotypic diversity among clinical isolates. The proteomic findings were not functionally validated, and a comprehensive safety assessment, including genomic analysis of transferable antibiotic resistance and virulence determinants, was not performed. Future studies should focus on mechanistic validation, expanded safety characterization, and inclusion of histological and immunological endpoints.

## 5 Conclusion

The pathogenicity and virulence of *H. pylori* infections include biofilm-forming, urease activity, and the ability to secrete toxins, such as CagA and VacA, which are associated with gastrointestinal inflammation and apoptosis. Moreover, the increasing antibiotic resistance in *H. pylori* underscores the need to develop alternative and adjuvant treatments to efficiently manage *H. pylori* infections.

Our results proved that *L. plantarum* L20 pH-adjusted CFS significantly inhibited the biofilm formation and the urease activity by downregulating the expression of urease enzyme and adhesion genes *cagA* and *vacA*. Furthermore, the L20 n-CFS reduced bacterial colonization in vivo. These findings suggest that *L. plantarum* L20-derived metabolites may represent a potential adjunctive strategy for *H. pylori* management; however, the results remain preliminary and do not support their use as a standalone therapeutic alternative. Further studies are required to confirm the underlying mechanisms, evaluate the interactions with conventional antibiotics, and establish the safety and efficacy through comprehensive preclinical and clinical investigations.

## Data Availability

The datasets presented in this study can be found in online repositories. The names of the repository/repositories and accession number(s) can be found at: http://www.proteomexchange.org/,PXD064835.
