# Supplementary material for: Novel anti-virulence strategy against Helicobacter pylori using Lactiplantibacillus plantarum cell-free supernatants
Source: Front Microbiol. 2026 Jul 3;17:1881796. doi: 10.3389/fmicb.2026.1881796 (PMC13376313; doi:10.3389/fmicb.2026.1881796)
Supplement: Supplementary file 1 [file Table_1.docx]

Supplementary Material

# Supplementary Figures and Tables

## Supplementary Tables

Supplementary Table 1. Antimicrobial susceptibility of lactic acid bacterial isolates against different antibiotics.

| **Sensitivity of the tested isolates against** | | | | | | | | | | | | | |
| --- | --- | --- | --- | --- | --- | --- | --- | --- | --- | --- | --- | --- | --- |
| **Isolate No.** | **Ampicillin** | **Penicillin** | **Cefotaxime** | **Erythromycin** | **Tetracycline** | **Gentamicin** | **Kanamycin** | **Streptomycin** | **Vancomycin** | **Clindamycin** | **Rifampin** | **Chloramphenicol** | **Trimethoprim** |
| **L12** | S | S | R | S | S | S | R | R | S | R | I | S | S |
| **L20** | S | S | I | S | S | S | R | I | R | S | S | S | S |
| **L22** | S | S | I | S | S | S | S | S | R | S | S | S | R |
| **L23** | S | S | R | S | S | R | R | R | R | S | S | S | R |
| **L27** | S | S | R | S | S | S | R | R | R | S | S | S | R |
| **L29** | S | S | R | S | S | R | R | R | R | S | S | S | R |
| **L32** | S | S | R | I | S | R | R | R | R | S | S | S | R |
| **L40** | S | S | R | S | S | S | R | I | R | S | S | S | S |
| **L51** | S | S | R | S | S | S | R | R | R | S | S | S | R |
| **L52** | S | S | R | R | S | S | R | R | R | S | S | S | S |
| **L54** | S | S | R | S | S | S | R | R | R | S | S | S | R |
| **L59** | S | S | R | S | I | S | R | R | R | S | S | S | R |
| **L92** | S | S | R | S | S | S | R | I | S | S | R | S | R |
| **L94** | S | S | R | S | I | S | R | R | R | S | S | S | R |
| **L100** | S | S | R | I | I | S | R | R | R | S | S | S | R |
| **L107** | I | S | R | I | S | S | R | R | R | S | S | S | R |
| **L109** | S | S | R | S | S | S | R | R | R | S | S | S | R |
| **L115** | S | S | R | I | S | S | R | R | S | S | R | S | R |
| **L118** | S | S | R | S | S | S | R | R | R | S | S | S | S |

Using the guidelines outlined in Materials and Methods, LAB isolates were categorized as either susceptible (S), intermediate (I), or resistant (R) to each antibiotic.

**Supplementary Table 2.** Total differentially identified proteins, including LC-MS/MS data, were classified according to their functions annotated in the database of Uniprot and GO annotations.

## Supplementary Figures


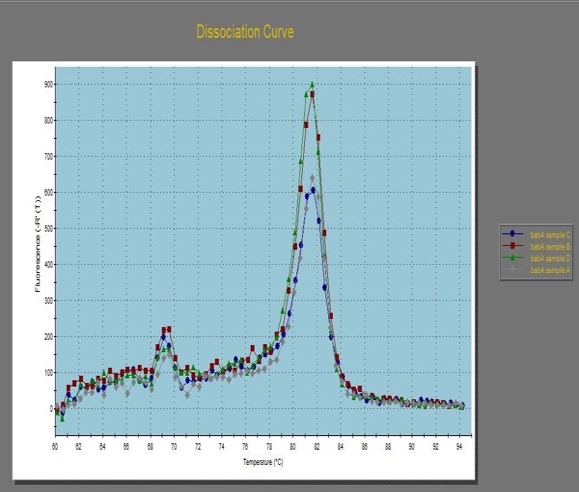

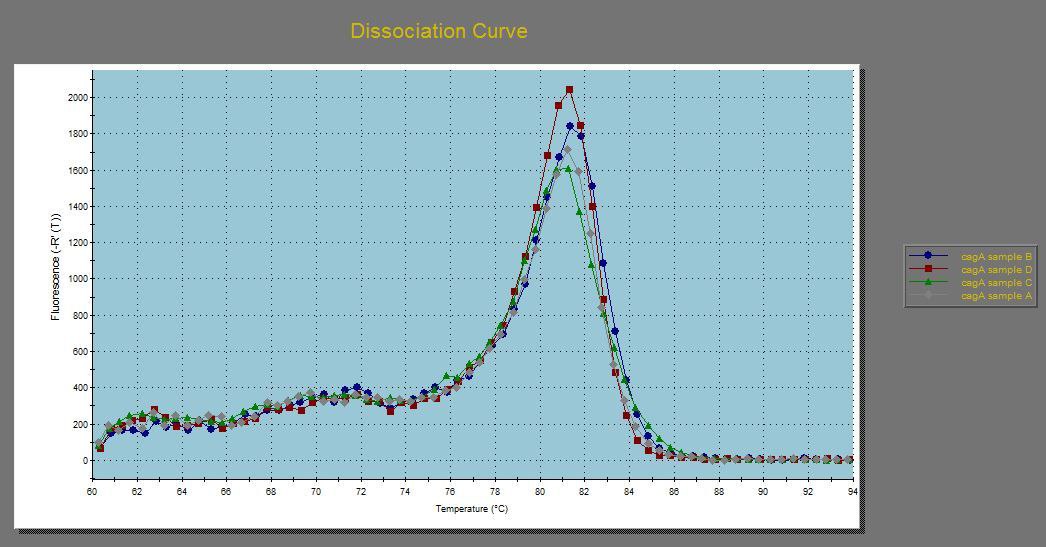

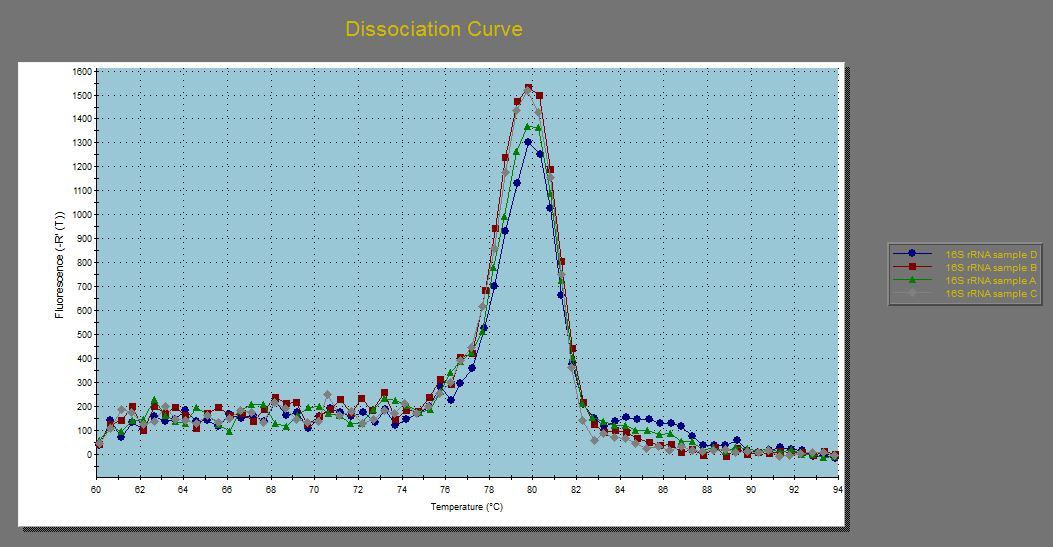

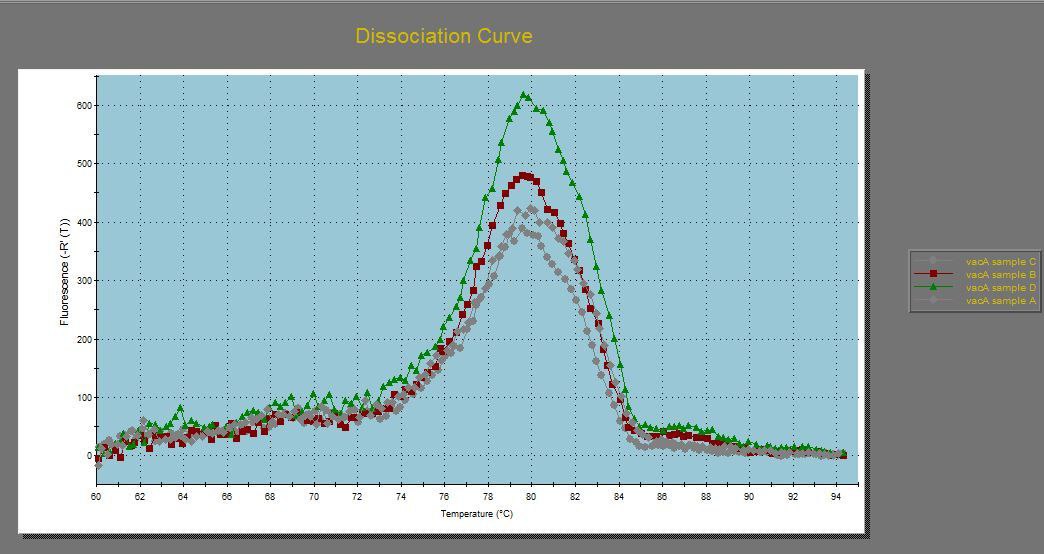

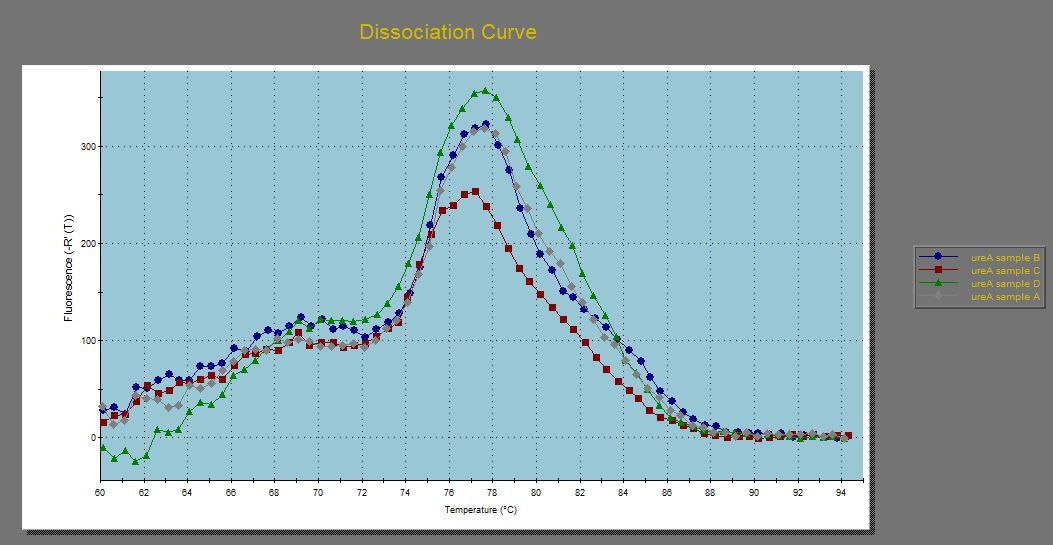


Supplementary Figure 1. Melting curve of the housekeeping gene (*16S rRNA*), *vacA*, *cagA*, *ureA*, and *babA* of the *H. pylori*.

Supplementary Figure 2. Growth curve of *H. pylori* in the presence of *L. plantarum* (L20 and L40) and *L. fermentum* L22. Growth was measured in the presence and absence of different n-CFS dilutions of *L.* plantarum L20, *L.* fermentum L22, and *L. plantarum* L40 at different incubation times from 0 to 48 h. The values were calculated as the average of three independent experiments, and the error bars represent the standard deviation. Significance (p < 0.05) was measured by using an unpaired Student’s t-test relative to the growth in BB supplemented with MRS broth.
